# Regulatory network reconstruction using an integral additive model with flexible kernel functions

**DOI:** 10.1186/1752-0509-2-8

**Published:** 2008-01-24

**Authors:** Eugene Novikov, Emmanuel Barillot

**Affiliations:** 1 Service Bioinformatique, Institut Curie, 26 Rue d'Ulm, 75248 Paris Cedex 05, France

## Abstract

**Background:**

Reconstruction of regulatory networks is one of the most challenging tasks of systems biology. A limited amount of experimental data and little prior knowledge make the problem difficult to solve. Although models that are currently used for inferring regulatory networks are sometimes able to make useful predictions about the structures and mechanisms of molecular interactions, there is still a strong demand to develop increasingly universal and accurate approaches for network reconstruction.

**Results:**

The additive regulation model is represented by a set of differential equations and is frequently used for network inference from time series data. Here we generalize this model by converting differential equations into integral equations with adjustable kernel functions. These kernel functions can be selected based on prior knowledge or defined through iterative improvement in data analysis. This makes the integral model very flexible and thus capable of covering a broad range of biological systems more adequately and specifically than previous models.

**Conclusion:**

We reconstructed network structures from artificial and real experimental data using differential and integral inference models. The artificial data were simulated using mathematical models implemented in JDesigner. The real data were publicly available yeast cell cycle microarray time series. The integral model outperformed the differential one for all cases. In the integral model, we tested the zero-degree polynomial and single exponential kernels. Further improvements could be expected if the kernel were selected more specifically depending on the system.

## Background

One of the most challenging tasks of systems biology is to reconstruct structures and mechanisms of interaction between components of cellular systems from available experimental data. In view of recent technological developments for large-scale measurements of DNA expression levels, this problem can often be formulated more specifically as a problem of gene network inference from microarray gene expression data. In particular, microarray time-series represent an important source of information about cellular dynamics. Various approaches have been proposed to reconstruct network structures from microarray time series. These approaches include additive regulation models [1,2], dynamic Bayesian networks (DBN) [3-5], S-system models [6,7] and Boolean networks [8,9]. Each of these concepts allows for several modifications, which multiplies the number of possible models for data analysis. The problem is not trivial as little is known about molecular interactions in experimentally observed systems. The mismatch between the real mechanisms used for data generation and the models used for network inference may lead to arbitrary network structures. Therefore it is difficult to expect that any one of the proposed formalizations can ensure acceptable performance for any biological system. Nevertheless further attempts to develop models that provide greater accuracy and flexibility with respect to the system under investigation would be appreciated.

The additive regulation model is a widely used approach for network inference from time series data [1]. It is represented by a set of ordinary differential equations:

$$\frac{dy_{i}(t)}{dt}=\sum_{j=1}^{n}w_{ij}y_{j}\left(t\right)+b_{i}$$

where *y*_*i*_(*t*) is the intensity level of node *i *at time *t*; *n *is the number of measured nodes; *b*_*i *_is the constant output observed in the absence of regularity inputs and *w*_*ij *_is the coefficient representing the influence of node *j *on the regulation of node *i*. As experimentally obtained time series are available in a finite number of discrete time points *N*, the continuous differential representation (1) should be converted into the discrete-time form:

$$y_{i}(t_{k+1})=y_{i}(t_{k})\left(1+w_{ii}\Delta t_{k}\right)+\sum_{\begin{array}{l} j=1\\ j\neq k \end{array}}^{n}w_{ij}y_{j}\left(t_{k}\right)\Delta t_{k}+b_{i}\Delta t_{k}$$

where *k = *1, ...,*N*-1 and Δ*t*_*k *_is the time interval between the measurements at times *t*_*k *_and *t*_*k*+1_.

Network inference fits developed models to experimental data. Fitting adjusts the unknown model parameters so that an optimal value for a fitness criterion is ensured. For the inference model(2), this criterion can be defined as

$$\chi^{2}=\frac{1}{Nn-P}{\sum_{i=1}^{n}\sum_{k=1}^{N}\frac{1}{\psi_{ik}^{2}}\left[{\hat{y}}_{i}(t_{k+1})-{\hat{y}}_{i}(t_{k})\left(1+w_{ii}\Delta t_{k}\right)-\sum_{\begin{array}{l} j=1\\ j\neq k \end{array}}^{n}w_{ij}{\hat{y}}_{j}\left(t_{k}\right)\Delta t_{k}-b_{i}\Delta t_{k}\right]}^{2}$$

where ${\hat{y}}_{i}$(*t*_*k*_) are the measured time series, *ψ *_*ik *_are the statistical weights and *P *is the number of estimated parameters. With the proper weights *ψ *_*ik*_, a *χ*^2 ^criterion value close to 1 indicates an acceptable fit. The estimated parameters encode information about the structure of the network.

In this paper we generalize the additive regulation model by converting differential equations into integral equations with adjustable kernel functions. These kernel functions can be selected based on prior knowledge or defined through iterative improvement in data analysis. This makes the integral model very flexible and thus capable of covering a broad range of biological systems more adequately and specifically than previous models. As the number of the unknown parameters for even medium-sized networks may exceed the number of experimentally measured points, fitting algorithms for underdetermined problems have to be applied. Among different fitting strategies [10] the forward selection fitting algorithm has shown reasonable performance, in particular for sparse networks, and, therefore, it has been adopted in this paper.

We tested the proposed generalization for the additive regulation model with simulated and experimental data. Mathematical models have been developed for real biological systems including the glycolysis pathway in yeast [11] and the mitogen-activated protein kinase (MAPK) cascade [12]. These models are available as SBML modules [13,14] that can be imported in JDesigner [15] to simulate time series. These time series are then sampled at random time intervals and statistical noise is added to mimic experimentally observed distortions. We also used the public yeast cell cycle microarray time series datasets measured by Spellman *et al*. [16] to demonstrate practical applicability of the developed approach.

## Results

### Mathematical Framework

The additive regulation model (1) can be easily used to derive first approximations for network structures. However, if the first-order ordinary differential equations (1) are not appropriate for a particular system or experimental dataset, the inference approach based on Eq. (1) provides little possibility for easy adjustments. Therefore we are looking for generalizations of the basic additive regulation model (1) that would allow us to systematically approximate broader range of dynamic behaviors. With this aim we integrate the ordinary differential equation (1) yielding:

$$y_{i}(t)-y_{i}(t_{0})=\sum_{j=1}^{n}w_{ij}\int_{t_{0}}^{t}y_{j}\left(t\right)dt+b_{i}\left(t-t_{0}\right)$$

where *t*_0 _is the initial time point. The coefficient *w*_*ij *_can be moved under the integral and converted into the function *w*_*ij*_(*t*, *x*):

$$y_{i}(t)=\sum_{j=1}^{n}\int_{t_{0}}^{t}w_{ij}\left(t,x\right)y_{j}\left(x\right)dx+b_{i}\left(t,t_{0}\right)$$

where *b*_*i*_(*t*, *t*_0_) is a function generalizing the second term in the right-hand part of Eq. (4). The fitness criterion for the integral model can be defined similar to Eq.(3):

$$\chi^{2}=\frac{1}{Nn-P}{\sum_{i=1}^{n}\sum_{k=1}^{N}\frac{1}{\psi_{ik}^{2}}\left[{\hat{y}}_{i}(t_{k})-\sum_{j=1}^{n}\int_{t_{0}}^{t_{k}}w_{ij}\left(t_{k},x\right){\hat{y}}_{j}\left(x\right)dx-b_{i}\left(t_{k},t_{0}\right)\right]}^{2}$$

Now the inference model is completely defined by the kernel functions *w*_*ij*_(*t*, *x*) and by the background functions *b*_*i*_(*t*, *t*_0_). This model, besides higher flexibility, allows for a straightforward interpretation in terms of control theory [17]. The integral equation (5) can be considered as the reaction of a system (gene *i*, in our case) on the *n *external inputs, represented by *y*_*i*_(*t*), with *w*_*ij*_(*t*, *x*) being system impulse response functions.

We propose the integral model (5) as a generic environment for devising more specific models. Instead of changing the form of the differential equation (which may lead to reprogramming of the inference algorithm), the integral model (5) allows for *continuous *change of the various parameters of the kernel or background functions. The parameters that are known from prior knowledge can be fixed in analysis, whereas the others can be made free and estimated from experimental data. Certain parameters can also be used to identify the shape of the kernel or background functions. Some examples of the generic representations for the kernel functions are given in the Methods section.

Higher model flexibility is accompanied by larger uncertainty about the derived structures, as different models or sets of model parameters can be in accordance with experimental data. Typical solutions for underdetermined problems are to collect more experimental data or to use more prior knowledge from the other sources of information. The advantage of the integral inference model is that (i) once we have more experimental data, we can leave more parameters free in fitting, and (ii) once we have more prior knowledge, we can smoothly integrate it in the inference model. In contrast, the differential model (2) needs to be redefined and reprogrammed in both cases.

The kernel or background functions can be rather complex for adequate description of the molecular/genetic interactions. As little has been formalized in this field so far, we have to use approximations. We are looking for such representations for *w*_*ij*_(*t*, *x*) and *b*_*i*_(*t*, *t*_0_) that result in the inference models linear with respect to the unknown parameters. These models can be represented as linear regression models allowing us to directly compute the best-fit parameters from the data. It is also straightforward to apply non-linear models, but these models lead to non-linear regression, requiring computationally intensive, iterative approaches. Therefore we generally prefer to use linear models unless we have strong evidence or prior knowledge that a model should be non-linear. Three linear models – polynomial, exponential and delta-function – for *w*_*ij*_(*t*, *x*) and *b*_*i*_(*t*, *t*_0_) are presented in the Methods section.

### Fitting Algorithm

The network reconstruction using the differential additive model (2) has been described in the Background section. The same approach can be applied for the developed integral model (5): this model is fit to experimental data and the unknown parameters are estimated by minimizing the *χ*^2 ^fitness criterion (6). Links created from the estimated parameters, if the corresponding parameters are significantly different from zero, form the network structure. In [10], different strategies to search for optimal network structures have been reviewed and compared. The searching strategies are model independent and therefore can be applied to both models, (2) and (5), without modification. Here we apply the forward selection algorithm [10] as a good compromise between prediction accuracy and speed of processing. The algorithm we use is essentially equivalent to the "Forw-reest-K" algorithm from [10]; we have just diversified a set of stopping criteria. The implemented algorithm is outlined as follows:

1. We begin without links for the network. A default model defined by Eq. (2) with all *w*_*ij *_= 0 or by Eq. (5) with all *w*_*ij*_(*t*, *x*) ≡ 0 is assigned to each non-interacting node.

2. The default model is fit to the data and the *χ*^2 ^fitness criterion is calculated for each node.

3. The node showing the largest *χ*^2 ^value is probably regulated by one of the other nodes. A link between the node of interest and each of the other nodes that are not yet identified as regulators for the node of interest is created.

4. The resulting sub-network is fit to the experimental data. The link that ensures the best quality of fit is conserved in the system.

5. The procedure generates links until the stopping criterion is fulfilled. We have implemented the following stop-criteria:

• We stop the procedure if the node with the lowest quality of fit is already linked to all the other nodes of the network. Thus, there are no more free nodes that can improve the fit for the node of interest (i.e. the node is saturated). This indicates that either the algorithm has achieved the local minimum or the inference model is not correct. In any case we still can continue to increase the overall quality of fit by more precise fitting for some of the other nodes. However, this may lead to over-fitting for these nodes and therefore is undesirable.

• The procedure can be stopped if the overall *χ*^2 ^quality criterion has reached a certain limit, or when the overall number of links (or the maximum number of links for one node) surpasses a user-defined value.

• Finally, the user can decide when to stop iterations based on visual inspection of the residuals – the differences between the experimental and the reconstructed time series. However, this may be problematic for large networks.

We use the *χ*^2 ^criterion as an indicator of correspondence between the inference model and experimental data because the inference model is expected to reproduce experimental data. However, if the statistical weights *ψ *_*ik *_in Eqs. (3) and (6) are not correct, the absolute value of the *χ*^2 ^criterion is meaningless. Using the experimental errors as *ψ *_*ik *_can lead to overestimation of *χ*^2^, because experimental data are presented in both the left- and the right-hand parts of the fitting models (2) or(5). Integration averages experimental errors in the right-hand part of Eq.(5). Thus, its contribution can be ignored in the overall statistical error, and *ψ *_*ik *_is equal to the experimental error. The sum in the right-hand part of Eq. (2) can also be considered as a smoothing operation. However, the error from the experimental point *y*_*i*_(*t*_*k*-1_) in the first term of the right-hand part of Eq. (2) is comparable to *y*_*i*_(*t*_*k*_) in the left-hand part of Eq. (2) and must be taken into account. In this case we define *ψ *_*ik *_as a product of experimental error and √2. Then values for *χ*^2 ^close to 1 indicate appropriate fit for both models.

If we assume that any link between any pair of nodes is possible, then the number of the unknown parameters can exceed the size of experimental datasets that are typically available. This leads to underdetermined systems and requires additional conditions to regularize the solution. In this respect the forward selection proceeds in a "natural", although not optimal, way: a new link is added only when it is necessary to increase the quality of fit.

The main problem of the algorithm is that it can easily be trapped in the local minima. If a wrong node is selected at an early iteration because it gives the best quality of fit for the selected node, the decision cannot be reconsidered at later iterations taking into account additional links created after that wrong decision. Nevertheless, we found that this algorithm performs reasonably well in many cases, particularly for relatively sparse networks.

### Testing

We compared performances of the differential and integral inference models using various artificial systems producing simulated data and three experimental datasets from [16].

As available experimental datasets are typically limited in size, we explored models where the number of free (fit) parameters was small. Thus we tested two kernels for the integral model: the zero-degree polynomial (*L*_*w *_= 0 in Eq. (8) and *L*_*b *_= 0 in Eq.(9)) and the single exponential (*L*_*w *_= 1 in Eq. (13) and *L*_*b *_= 0 in Eq.(14)). In each case we had one free parameter per link. This also equalizes the degrees of freedom in the compared differential and integral inference models. The delta-function model described in the Methods section was not applied because all tested systems demonstrate behavior continuous in time.

To appreciate how our predictions are far from random, we also applied the integral model with the zero-degree polynomial kernel to infer network structures from the permuted data, i.e. when node labels are randomly assigned to generated time series.

### Arbitrary Networks

In the first set of experiments the model used for network inference was that used for data generation.

#### Simulation

**Figure 1:**
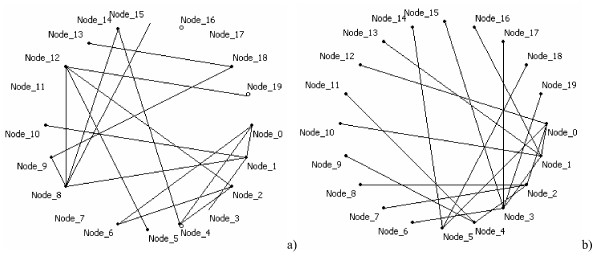
**Examples of 20-node artificial networks**. Network topology: (a) random with *p *= 0.05 and (b) scale free with *γ *= 2.5.

Artificial regulatory networks were generated with random and scale-free topologies. For random topology, any two nodes are connected with the probability *p *independent from the other connections. For scale-free topology [18], the number of links at each node is approximated by a power-law distribution *p*(*k*) ~ *k*^γ ^. We used the *growing network with redirection *algorithm [19] to generate networks with scale-free topology. The number of nodes in the generated networks was 20; the probability *p *for the random networks was equal to 0.05; and the parameter *γ *for the scale-free networks was set to 2.5 for all cases. We demonstrate examples of networks undergoing random topology (Fig. 1a) and scale-free topology (Fig. 1b). A set of first-order ordinary differential equations (1) was used to simulate time series. The parameters *w*_*ij *_were randomly generated from the uniform distribution in the interval [-1;1]. The background levels *b*_*i *_were set to zero and the initial states *y*_*i*_(*t*_0_) were set to 1 for all nodes.

We used the fourth-order Runge-Kutta formula [20] to numerically solve differential equations(1). The solution was built on 1000 time points uniformly spaced over the interval [0;10]. The resulting time series were sampled to produce 20 time points to approach the quality of experimental data. We split the original 1000-point time series into 20 intervals of 50 points. At each interval the output time point was randomly selected. This led to a time series with non-homogeneous (random) time intervals between subsequent measurements. Each of 20 intensity values was statistically distorted. The distorted value was generated as a Gaussian random variable with the mean equal to the true value and standard deviation proportional to the true value. The coefficient of proportionality – noise-to-signal level – was set to 0.05.

#### Inference

As time series were simulated using a set of first-order ordinary differential equations, the corresponding inference model is either the differential model (Eq. (2)) or the integral model (Eq.(5)) with the zero-degree polynomial kernel (*L*_*w *_= 0 in Eq. (8) and *L*_*b *_= 0 in Eq.(9)). Although the single exponential kernel may also be used in this case, it is clearly non-adequate and therefore it was not tested.

We reconstructed the networks from the generated time series using the forward selection procedure. Each time the fitting procedure added a new link, we updated the number of links for True Positives (*TP*), False Positives (*FP*) and False Negatives (*FN*). Then *TP*, *FP *and *FN *values were combined to estimate Positive Predictive Value (PPV) and Sensitivity value (Se) defined as in [21]:

$$\begin{array}{cc} PPV=\frac{TP}{TP+FP}; & Se=\frac{TP}{TP+FN} \end{array}$$

Other possible performance measures, such as negative predictive value or specificity, are not relevant for sparse networks when the forward selection procedure is used for reconstruction. During first iterations of the fitting procedure the number of *TN *largely exceeds the number of *TP *leveling the difference between reconstruction models.

We stopped the forward selection procedure if the *χ*^2 ^fitting criterion became smaller than 0.5 or if a particular node became saturated. Adequate fit should give *χ*^2 ^values close to 1, as experimental errors – and thus statistical weights *ψ *_*ik *_– in the *χ*^2 ^criteria for Eqs. (3) or (6) are directly accessible in simulations. Limiting the value of the *χ*^2 ^criterion to 0.5 leads to substantial over-fitting. However, as we recorded the history of generated links (PPV, Se and *χ*^2 ^value after each added link), this allowed us to explore a broader range of model fitness values.

We averaged the dependence of PPV and Se on the total number of links over 100 runs of the simulation procedure. A different network structure, different link parameters, different time sampling and different noise realizations were generated at each run.

### Artificial Biological Systems

We used two mathematical models for real biological systems (yeast glycolysis [11] and the MAPK cascade [12]) to test the performance of the developed inference models for more realistic systems. These models can be imported in JDesigner [15] as SBML modules [13,14] and used to simulate time series. The network structures and SBML files used for simulations are also available from our web page [22]. We stress that we used these modules as they were originally developed, i.e. without any modifications in the structure or in the kinetic parameters of the models. Mathematical representations and kinetic parameters of the models can be viewed in JDesigner. We used JDesigner to integrate the models on 100 time points spaced uniformly over the interval [0;1] for yeast glycolysis and [0;100] for the MAPK cascade.

Two data distorting steps were performed as before: we left 20 time points at random time intervals, and added Gaussian noise with noise-to-signal level equal to 0.05. Examples of time series used for the inference are available on our web page [22].

Besides comparing the differential and integral inference models, we also tested here two kernels for the integral model: the zero-degree polynomial (*L*_*w *_= 0 in Eq. (8) and *L*_*b *_= 0 in Eq.(9)) and single exponential (*L*_*w *_= 1 in Eq. (13) and *L*_*b *_= 0 in Eq.(14)).

The forward selection fitting procedure generated the dependence of the PPV, Se (Eq.(7)) and *χ*^2 ^criteria (Eqs. (3) and(6)) on the total number of generated links. The resulting curves were averaged over 100 runs of the simulation procedure. The simulation procedure generated different time sampling and different realizations of noise at each run, whereas the network structure, kinetic laws and kinetic parameters remained the same.

### Real Data

To demonstrate applicability of the developed approach to real experimental data, we used the yeast (*Saccharomyces cerevisiae*) cell cycle microarray time series dataset [16]. This dataset consists of three sub-sets measured using different cells synchronization methods [16]: α factor-based (*alpha*, 18 time points), size-based (*elu*, 14 time points) and cdc15-based (*cdc15*, 24 time points).

**Figure 2:**
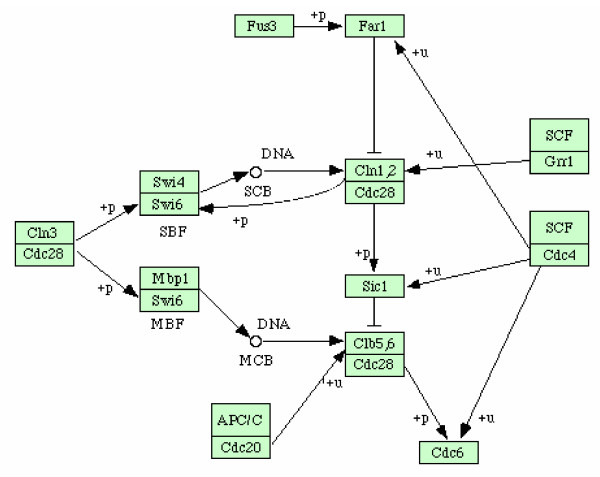
A part of the yeast cell cycle pathway available from KEGG [26].

As others did [23-25], we selected a part of the yeast cell cycle pathway available from KEGG [26] (Fig. 2). Assuming that this pathway reflects biological reality, we can count the number of *TP*, *FP *and *FN *and calculate PPV and Se as it is done for artificial systems.

As experimental errors and therefore the statistical weights *ψ *_*ik *_in Eqs. (3) or (6) were not available, the absolute value of the *χ*^2 ^fitting criterion could not be used as a stopping condition for the forward selection procedure. However, as it will be shown for artificial systems (see the Discussion section), numerous *FP *links are required to yield the *χ*^2 ^criterion close to 1. Taking into account that fitting models are very approximate, it may not be always reasonable to require perfect fitting quality. Therefore we investigated the performance (PPV and Se) of the inference models as a function of the number of generated links.

As for the artificial systems, we compared here performances of the differential and integral inference models. In the integral model we used the same two kernels: the zero-degree polynomial (*L*_*w *_= 0 in Eq. (8) and *L*_*b *_= 0 in Eq.(9)) and single exponential (*L*_*w *_= 1 in Eq. (13) and *L*_*b *_= 0 in Eq.(14)).

We also applied DBN approach to infer network structures from the experimental datasets. We used the Banjo software [27] to perform Bayesian inference. For analysis, we selected the *alpha *and *elu *datasets as only these two datasets were measured at equidistant time points. The latter is prerequisite for Banjo. To run Banjo we used the same input settings as given in [21]. We calculated PPV and Se for the inferred networks that had the highest score in the Banjo output.

### Independent artificial data

Finally, we performed an additional comparison of the differential and integral inference models based on an independent set of artificial data described in [21]. Briefly, 20 random 10-gene networks with an average in-degree per gene of 2 were generated. For each network, time-series data (1000 time points) were simulated using linear ordinary differential equations. Each data point was statistically distorted with noise-to-signal ratio equal to 0.1. In our analysis we first sampled the 1000-point time series to produce 20-point time series, which were then used for network reconstruction. As the network structures are known, we built the dependencies of PPV and Se on the number of generated links for each network. The obtained dependencies were further averaged over 20 networks.

### Software

The developed algorithms for the network inference were implemented in the software package NETI, freely available from our web page [22].

## Discussion

### Arbitrary Networks

**Figure 3:**
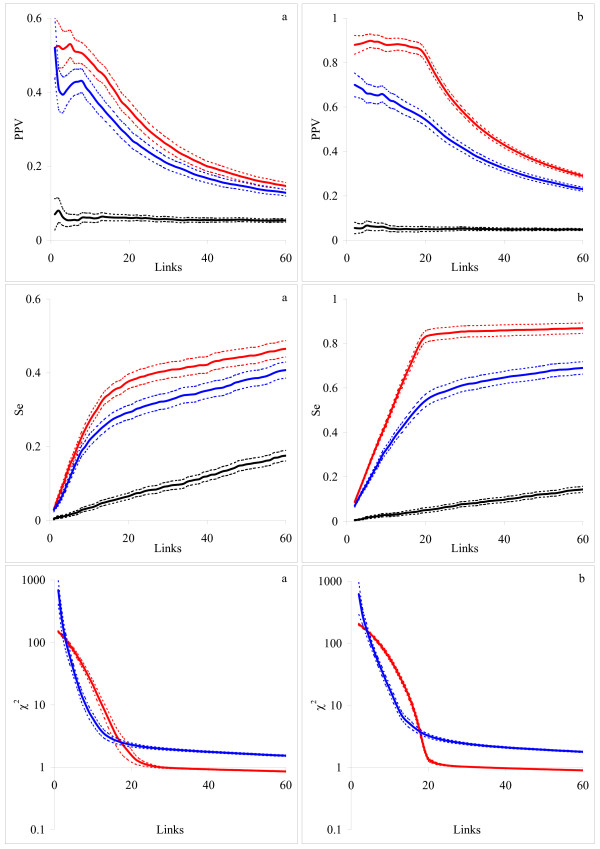
**The average dependencies of PPV, Se and *χ*^2 ^criterion on the total number of links for arbitrary networks**. Network topology: (a) random and (b) scale free. Inference models: differential (blue) and integral with the zero-degree polynomial kernel (red). The black lines indicate the inference by the integral model with the zero-degree polynomial kernel from permuted data. Confidence intervals for the obtained PPV, Se and *χ*^2 ^estimates are shown as dashed lines.

We present the resulting PPV and Se curves for random topologies (Fig. 3a) and scale-free topologies (Fig. 3b). We also show the dependence of the averaged overall fitness (*χ*^2^) on the number of links. The *χ*^2 ^criteria were calculated from Eq. (3) for the differential inference model and from Eq. (6) for the integral inference model. We found that the integral model was superior to the differential model for both scale-free and random topologies, demonstrating higher predictive power and sensitivity. The networks with scale-free topology were reconstructed with greater accuracy (i.e. with smaller number of *FP *and *FN*) than those with random topology. Moreover, adequate fit (*χ*^2 ^is close to 1) corresponded to the best reconstruction (the highest PPV) only for the scale-free networks. For random topology the best reconstruction was achieved at a *χ*^2 ^value somewhat greater than 1. In this case, the inference procedure needed more links to reproduce the simulated time series. Many of those links were false positives, decreasing the PPV values. The better performance for the scale-free networks can be due to the fact that they had fewer nodes that simultaneously regulated another node. Therefore, the fitting procedure has fewer chances to incorrectly select a node as a regulator. Despite the correspondence between data producing models and network inference models, reconstruction was not perfect. There are various reasons for that.

Although the underlying mathematical models were equivalent, the numerical implementations were different. We used an algorithm based on the fourth-order Runge-Kutta formula for data generation. This was more accurate than the algorithms that we used for reconstruction: simple Euler formula [20] in the differential model or trapezoidal rule (Eqs. (12) or(16)) in the integral approach. As the Euler formula is less numerically accurate than the trapezoidal rule, the differential model may generate more false positives.

Randomized time sampling and statistical distortions further reduced the accuracy of reconstruction. However, we expect that the integral model should be more resistant to noise, as each data point is approximated by an integral (Eq.(5)), smoothing noise contribution from all previous data points. In the differential model, the only one, previous, time point is used to fit the current one, and therefore the recovered values are subject to higher variation.

Model identifiability is another problem: even if we could collect an infinite amount of experimental data and implement an "ideal" fitting procedure [28], the model might not be identifiable for certain network configurations. A model might become non-identifiable if, for example, two nodes demonstrate (by chance) similar behavior, and are indistinguishable under realistic noise-to-signal levels and/or with numerical errors.

Finally, we note that non-perfect performance of the fitting procedure can lead to local minima solutions.

### Artificial Biological Systems

**Figure 4:**
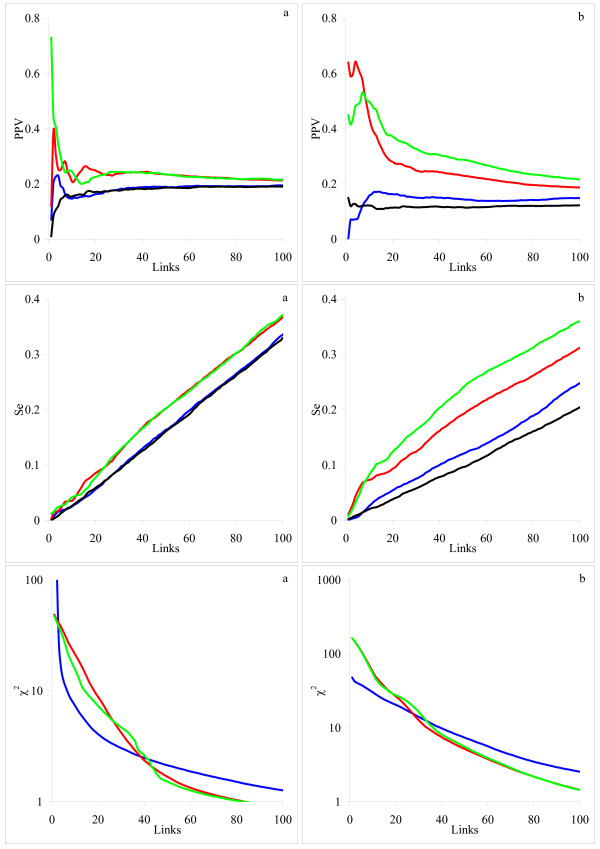
**The average dependencies of PPV, Se and *χ*^2 ^criterion on the total number of links for two artificial biological systems**. System: (a) yeast glycolysis and (b) MAPK. Inference models: differential (blue), integral with the zero-degree polynomial kernel (red) and integral with the single-exponential kernel (green). The black lines indicate the inference by the integral model with the zero-degree polynomial kernel from permuted data. Confidence intervals for the obtained estimates are too narrow to be recognizable in the graphs and therefore not shown.

Our results demonstrate the advantage of the integral inference model for both artificial biological systems: the yeast glycolysis pathway (Fig. 4a) and the MAPK cascade (Fig. 4b). However, this approach did not perform as well as in the case when a set of linear differential equations was used to generate data. This is due to inadequacy of the model used for the network inference to that used for data generation. This model inadequacy is also the reason why the inference model needs so many links to reproduce the simulated time series reasonably well (*χ*^2^*≅ *1). Good approximation corresponds to very modest PPV, whereas the highest PPV was achieved at a much larger *χ*^2 ^value with fewer links. Our findings indicate that the links generated during early stages of network reconstruction are more accurate than those generated later. Links generated later may be needed only to improve approximation.

Comparing the zero-degree polynomial and single exponential kernels, neither showed clear advantage. Moreover, their performance differed depending on the number of generated links. For the region with the highest PPV (< 10 links), the polynomial kernel seemed to be more powerful for the MAPK cascade (Fig. 4b), whereas the exponential kernel gave better results for the yeast glycolysis pathway (Fig. 4a). This poses an important problem of adequate selection of the kernel function. Different ways, ranging from formalizing prior knowledge to more elaborated algorithms of model fitting, can be envisaged. This, however, remains a subject of future work.

### Real Data

**Figure 5:**
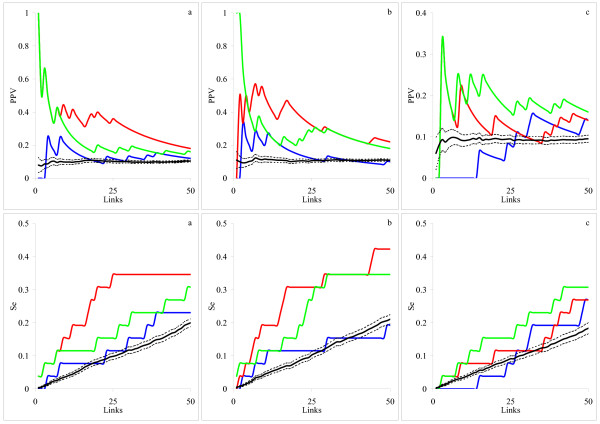
**The dependencies of PPV and Se on the total number of links for the three yeast cell cycle microarray time series datasets**. Synchronization method: (a) *alpha*, (b) *elu *and (c) *cdc15*. Inference models: differential (blue), integral with the zero-degree polynomial kernel (red) and integral with the single-exponential kernel (green). The black lines indicate the average over 100 random permutations dependencies of PPV and Se for the integral model with the zero-degree polynomial kernel. Confidence intervals for the permuted PPV and Se are shown as dashed lines.

From the resulting PPV and Se curves presented in Fig. 5 we conclude that the integral model with either polynomial or exponential kernels outperforms the differential model for all three experimental sub-sets. The reconstruction models showed similar performance for the *alpha *and *elu *experimental datasets, whereas for the *cdc15 *set, PPV and Se values were somewhat lower. This suggests that a different, more adequate, model should be found in that case.

As for the artificial systems, there was no systematic advantage of one integral kernel versus another one. The polynomial kernel generally produced higher PPV and Se values for the *alpha *and *elu *experimental datasets (Fig 5a, b), and the exponential kernel was more performing for the *cdc15 *(Fig. 5c). These observations confirm a conclusion that adequate kernel selection may lead to substantial improvements in the reconstruction.

In the Banjo output, the highest score networks had 37 links for the *alpha *dataset and 41 links for the *elu *dataset. We compared the DBN performance with performances shown by the differential and integral (with polynomial and exponential kernels) additive models. As the forward selection algorithm built dependencies of PPV and Se on the number of generated links, we selected PPV and Se at the same number of links as generated by Banjo (37 for the *alpha *dataset and 41 for the *elu *dataset). The results are collected in Table 1. We can conclude that the DBN performance is comparable to the performance of the differential inference model and both are outperformed by the integral inference model with either polynomial or exponential kernels. Polynomial kernel is the most powerful for the *alpha *dataset at the given number of links. These results should be considered with caution as applying inference models is conditioned on the algorithm of reconstruction: simulated annealing in Banjo and forward selection for the integral additive model. As a subject for further research, it may be promising to implement the integral additive model in the DBN framework.

**Table 1:** PPV and Se of the network reconstruction using the DBN approach, differential (A) and integral (with the polynomial (B) and exponential (C) kernels) inference models. The number of generated links was 37 for the *alpha *dataset and 41 for the *elu *dataset.

		DBN	A	B	C	B*
*alpha*	PPV	0.128	0.135	0.243	0.162	0.097 ± 0.008
	Se	0.169	0.192	0.346	0.231	0.138 ± 0.011
*elu*	PPV	0.098	0.098	0.220	0.220	0.108 ± 0.008
	Se	0.174	0.154	0.346	0.346	0.170 ± 0.012

*) reconstruction from 100 random permutations.

### Independent artificial data

**Figure 6:**
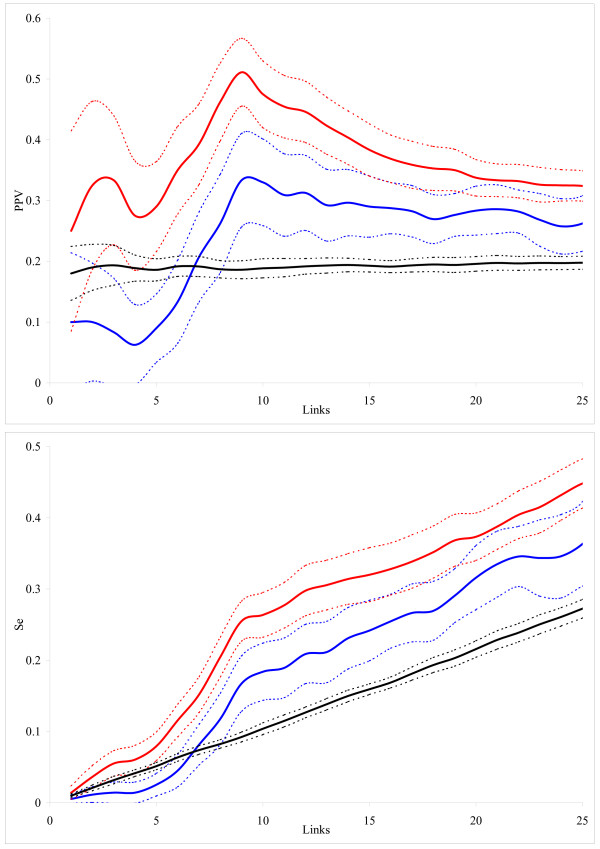
**The average dependence of PPV and Se on the total number of links for the independent set of artificial data [21]**. Inference models: differential (blue) and integral with the zero-degree polynomial kernel (red). The black line indicates the inference by the integral model with the zero-degree polynomial kernel from permuted data. Confidence intervals for the obtained estimates are shown as dashed lines.

The average dependencies of PPV and Se on the number of generated links are presented in Fig. 6. As for our own artificial data (Fig 3), the integral inference model demonstrated clear advantage for the independent dataset too. We note three differences as compared to Fig. 3: (i) the number of generated links in Fig 6 is smaller because the networks are smaller in the independent dataset (10 nodes network in Fig. 6 against 20 nodes network in Fig. 3), (ii) the confidence intervals in Fig. 6 are wider because the number of available networks is smaller in the independent dataset (10 networks in Fig. 6 against 100 networks in Fig. 3), and (iii) as in [21], we did not include self-feedback loops when computing PPV and Se, although those are presented in the network structure (diagonal elements of the adjacency matrix). The latter might lead to decreased predictive power as the both, differential and integral, inference models can account for self-regulation.

To summarize the obtained results, we note that although the performance of the integral inference model differed depending on the system, it was always superior to the differential inference model. In the integral model, we used the zero-degree polynomial kernel and the single-exponential kernel with the fixed decay time. The decay time (0.9*T*, where *T *is the last time point in a time series) was selected such that the kernel function decreased slowly within the measurement time range. The zero-degree polynomial kernel can also be considered as a particular case of the exponential kernel with the decay time approaching infinity or, in practical applications, just somewhat bigger than *T*. Therefore the variation between two kernels was not expected to largely influence the performance. However, the observed difference in the inference results was sometimes significant (for example, Fig. 4b, or Fig. 5). This indicates that refined selection of the kernel function can be an important perspective for network inference improvements.

## Conclusion

In this paper we propose a generalization of the additive regulation model represented by a set of differential equations(1). Differential equations are one of the well-advanced formalizations in biochemical systems modeling. Although the model defined by Eq. (1) is a rough approximation, it can be progressively modified to cover more realistic models that adequately account for interaction mechanisms and kinetic rates.

One way to increase flexibility of this model is to convert it into a set of integral equations with adjustable kernel functions. Then, instead of changing the form of the differential equation, changing the kernel function or the various parameters of the kernel function allows the model to cover a broad range of systems. Properly identifying the kernel function can make the inference model more specific for the system under investigation and ensure improved accuracy of network reconstruction. Thus, our proposed approach is a generalization in a sense that it provides an easy and broadly applicable way to create specific models for particular datasets. The model can be adjusted by parametric fitting, using complimentary experimental data and by formalizing knowledge from the literature and biological databases.

The basic model that we consider in this paper is additive, i.e. the cooperative regulatory contribution of different nodes is a sum of the contributions from each node. Integral representation can also cover more complicated schemes including an S-system model [6,7], defined as a set of zero-degree ordinary differential equations with higher-order kinetic rate laws.

The integral inference model (5) can be incorporated into the DBN framework in the same way as it was suggested for the differential model (1) [29,30]. In this case such equations as (11) or (15) can be used to specify conditional links between the nodes and the corresponding conditional probability distributions.

In this paper, the kernel functions (zero-degree polynomial and single exponential with the fixed decay time) were characterized by a single unknown parameter per link. Consequently the compared integral and differential inference models had the same overall number of degrees of freedom. Thus, just by reshaping the inference model, while preserving the total number of free parameters, we were able to improve network reconstruction. However, the problem of network inference may often be underdetermined even for such simplified models: the number of the unknown parameters may exceed the number of experimentally measured points. Although the forward selection fitting algorithm offers an effective solution to the problem, it is not the only possible approach. For example, interpolation techniques [2,31] can be used to artificially increase the amount of experimental data, or dimensional reduction methods [2,32] can be used to decrease the number of free parameters. As more complicated inference models, characterized by larger number of parameters, can be envisaged, the choice of the most appropriate approach for solving underdetermined problems deserves special attention in the future.

The forward selection fitting algorithm can be improved as well. For example, as considered in [10], we can explore regulatory nodes by pairs, triples, etc. rather than one by one. This might avoid the local minima problem, but would definitely increase the time of processing. Though adding nodes one by one is not a perfect solution, it creates the dependence of the model performance (PPV and Se values) on the number of generated links. The importance and trustworthiness of the generated links are functions of iteration of the forward selection procedure that generated these links. The links generated during early stages of reconstruction should gain more attention in the follow up analysis. This approach releases importance of the stopping criteria; which, for real experimental data, are often difficult to formulate.

Networks derived from limited data should only be considered as rough approximations for real network structures. Experiments should be designed to yield datasets to improve the reconstruction. Therefore, *reverse engineering *of the regulatory networks should be defined as an iterative process where the steps of *network inference *and *experimental design *are performed in turn. Thus, the initially derived network can be used to optimally design experiments. This would allow improved identification of the network structure with less experimental effort and expense. Proper formalization of such iterative algorithm is a subject of further research.

## Methods

We have developed three representations – polynomial, exponential and delta-function – for *w*_*ij*_(*t*, *x*) and *b*_*i*_(*t*, *t*_0_) resulting in the inference models linear with respect to the unknown parameters.

### Polynomial Model

The polynomial model is given by

$$w_{ij}\left(t,x\right)=\sum_{l=0}^{L_{w}}u_{l,ij}{\left(t-x\right)}^{l}$$

$$b_{i}\left(t,t_{0}\right)=\sum_{l=0}^{L_{b}}v_{l,i}{\left(t-t_{0}\right)}^{l}$$

where *u*_*l*,*ij*_, *l *= 0, ...,*L*_*w *_are the polynomial coefficients approximating the influence of node *j *on node *i *and *v*_*l*,*i*_, *l *= 0, ...*L*_*b *_are the background polynomial coefficients. Substituting Eq. (8) in the integral in the right-hand side of Eq. (5) yields

$$\int_{t_{0}}^{t}w_{ij}\left(t,x\right)y_{j}\left(x\right)dx=\sum_{l=0}^{L_{w}}u_{l,ij}\int_{0}^{t}{\left(t-x\right)}^{l}y_{j}\left(x\right)dx=\sum_{l=0}^{L_{w}}u_{l,ij}\sum_{m=0}^{l}C_{l}^{m}t^{m}\int_{0}^{t}{\left(-x\right)}^{l-m}y_{j}\left(x\right)dx$$

where $C_{l}^{m}$ are the binomial coefficients. If we substitute Eq. (10) in Eq. (5) and then convert the resulting equation into discrete-time representation, we obtain

$$y_{i}(t_{k})=\sum_{j=1}^{n}\sum_{l=0}^{L_{w}}u_{l,ij}\sum_{m=0}^{l}C_{l}^{m}t^{m}I_{j,l-m}\left(t_{k}\right)+\sum_{l=0}^{L_{b}}v_{l,i}{\left(t_{k}-t_{0}\right)}^{l}$$

where *I*_*j*,*l*-*m*_(*t*_*k*_) is calculated recurrently using the trapezoidal rule for integration [20]:

$$I_{j,l-m}\left(t_{k}\right)=I_{j,l-m}\left(t_{k-1}\right)+\frac{{\left(-t_{k}\right)}^{l-m}y_{j}\left(t_{k}\right)+{\left(-t_{k-1}\right)}^{l-m}y_{j}\left(t_{k-1}\right)}{2}\Delta t_{k}$$

### Exponential Model

We define the exponential model as a sum of exponentials:

$$w_{ij}\left(t,x\right)=\sum_{l=1}^{L_{w}}u_{l,ij}\exp\left\{-\left(t-x\right)/\tau_{l,ij}\right\}$$

$$b_{i}\left(t,t_{0}\right)=v_{0i}+\sum_{l=1}^{L_{b}}v_{l,i}\exp\left\{-\left(t-t_{0}\right)/\lambda_{l,i}\right\}$$

where *u*_*l*,*ij *_are the exponential amplitudes and *τ *_*l*,*ij *_are the decay times approximating the influence of node *j *on node *i *(*l *= 1, ...,*L*_*w*_), *v*_0,*i *_and *v*_*l*,*i *_are the background exponential amplitudes and *λ *_*l*,*i *_are the background decay times (*l *= 1, ...*L*_*b*_). With the *L*_*w*_-exponential *w*_*ij*_(*t*, *x*), the integral equation (5) can be converted into a *L*_*w*_-order ordinary differential equation (e.g. in [29] or in [31] for *L*_*w *_= 2).

The exponential decay times, *τ *_*l*,*ij *_and *λ *_*l*,*i*_, and the exponential amplitudes, *u*_*l*,*ij *_and *v*_*l*,*i*_, can be fit. However, this will lead to models that are non-linear with respect to the unknown parameters. We can assume that *τ *_*l*,*ij *_and *λ *_*l*,*i *_are independent of the nodes (*τ *_*l*,*ij *_= *τ *_*l *_and *λ *_*l*,*i *_= *λ *_*l*_) and then fix *τ *_*l *_and *λ *_*l *_during the fit. Once *τ *_*l *_and *λ *_*l *_are fixed, we need to estimate only the exponential amplitudes *u*_*l*,*ij *_and *v*_*l*,*i*_.

In this paper, for example, we always used the constant background (*L*_*b *_= 0 in Eq.(14)) and the single-exponential kernel (*L*_*w *_= 1 in Eq.(13)) with *τ *_0 _= 0.9*T*, where *T *is the last time point in a time series. This decay time approximates relatively slow processes occurring in the system. Note that with further increase of *τ *_0_, the single-exponential kernel will approximate the zero-degree polynomial kernel more precisely. We selected *τ *_0 _= 0.9*T *to test if a relatively small variation of the kernel function (as compared to the zero-degree polynomial) could significantly influence network reconstruction.

Substituting Eqs. (13) and (14) in Eq. (5) and then converting the resulting expression into discrete-time form yields

$$y_{i}(t_{k})=\sum_{j=1}^{n}\sum_{l=1}^{L_{w}}u_{l,ij}I_{j,l}\left(t_{k}\right)+v_{0i}+\sum_{l=1}^{L_{b}}v_{l,i}\exp\left\{-\left(t_{k}-t_{0}\right)/\lambda_{l,i}\right\}$$

where *I*_*l*_(*t*_*k*_) is calculated recurrently using the trapezoidal rule for integration [20]:

$$I_{j,l}\left(t_{k}\right)=\exp\left\{-\Delta t_{k}/\tau_{l}\right\}I_{j,l}\left(t_{k-1}\right)+\frac{y_{j}\left(t_{k}\right)+\exp\left\{-\Delta t_{k}/\tau_{l}\right\}y_{j}\left(t_{k-1}\right)}{2}\Delta t_{k}$$

### Delta-function Model

The delta-function model can be represented as

$$w_{ij}\left(t,x\right)=\sum_{l=0}^{L_{w}}u_{l,ij}\delta \left\{t-\left(x-\mu_{l,ij}\right)\right\}$$

*b*_*i *_(*t*, *t*_0_) = *v*_0*i*_

where *u*_*l*,*ij *_are the weights of contribution from the previous time points *x-μ*_*l*,*ij *_to the current one *t *(*l *= 0, ...,*L*_*w*_), for the regulation of node *i *by node *j*; *μ*_*l*,*ij *_are the time delays and *v*_0,*i *_is the background level. This model explicitly takes into account time delays in regulation. The integral equation (5) with the delta-function kernel (17) can be transformed into a difference equation similar to [33].

### Combined Model

In general, we can always define kernel/background functions as algebraic combinations of different elementary functions. An example of such generalized kernel can be represented as a sum of the polynomial (8) and exponential (13) models:

$$w_{ij}\left(t,x\right)=y^{P}\sum_{l=0}^{L_{w}^{P}}u_{l,ij}^{P}{\left(t-x\right)}^{l}+y^{E}\sum_{l=1}^{L_{w}^{E}}u_{l,ij}^{E}\exp\left\{-\left(t-x\right)/\tau_{l,ij}\right\}$$

where *P *and *E *designate parameters of the polynomial and exponential models, respectively, and *y*^*P *^and *y*^*E *^define the weight of each elementary model into the overall kernel function. If *y*^*P *^and *y*^*E *^are unknown and can not be fixed during the fit, they should be incorporated in the fit parameters $u_{l,ij}^{P}$ and $u_{l,ij}^{E}$.

## Authors' contributions

EN developed the model, performed software implementation and drafted the manuscript. EB conceived of the study and participated in coordination. All authors read and approved the final manuscript.
